# Association of Sugar-Sweetened Beverage Frequency with Adiposity: Evidence from the “Children of 1997” Birth Cohort

**DOI:** 10.3390/nu12041015

**Published:** 2020-04-07

**Authors:** Ting Zhang, Shiu Lun Au Yeung, Man Ki Kwok, Lai Ling Hui, Gabriel Matthew Leung, C. Mary Schooling

**Affiliations:** 1 School of Public Health, Li Ka Shing Faculty of Medicine, The University of Hong Kong, Hong Kong SAR, China; zhangt77@connect.hku.hk (T.Z.); ryanaysl@connect.hku.hk (S.L.A.Y.); maggiek@hku.hk (M.K.K.); huic@hku.hk (L.L.H.); gmleung@hku.hk (G.M.L.); 2 Department of Pediatrics, Faculty of Medicine, The Chinese University of Hong Kong, Hong Kong SAR, China; 3 CUNY School of Public Health and Health Policy, New York, NY 10027, USA

**Keywords:** adiposity, children, sugar-sweetened beverages

## Abstract

Background: Observationally, sugar-sweetened beverage (SSB) consumption is associated with adiposity in Western children but could be confounded. We examined the association of SSB frequency with adiposity in the non-Western setting of Hong Kong. Methods: We examined the associations of SSB consumption frequency at 11 and 13 years assessed by using a food frequency questionnaire with subsequent body mass index (BMI) z-score and overweight/obesity up to 18 years using generalized estimating equations, and with waist circumference, waist-to-hip ratio, and body fat percentage at 16–19 years using linear regression in a population-representative Chinese birth cohort “Children of 1997” (*n* = 3628). Results: At 11 and 13 years, 6.8% and 8.2% of children respectively consumed SSB daily. Neither SSB frequency at 11 nor at 13 years was associated with subsequent BMI z-score or overweight/obesity up to 18 years, or with waist circumference, waist-to-hip ratio, or body fat percentage at 16–19 years adjusted for age, sex, socioeconomic position, health status, physical activity and other food consumption, although bias to the null from under-reporting cannot be eliminated. Conclusion: Although we cannot definitively exclude a small association of SSB frequency with adiposity, lack of association of SSB frequency with adiposity in a non-Western setting with low SSB consumption suggests that the role of SSB in adiposity appears to be minor.

## 1. Background

Mean body mass index (BMI) and the prevalence of obesity in children and adolescents increased dramatically from 1975 to 2016 [1]. Added sugars, particularly in the form of sugar-sweetened beverages (SSB), contribute to excess calories, and may play a role in the development of obesity, because sugars in liquid form may induce less satiety than in solid form [2] and promote the over-consumption of calories. Health organizations, including the World Health Organization (WHO) and the American Heart Association, have recommended restricting SSB intake to address the obesity epidemic [3,4]. Meta-analyses of observational studies mainly from Western settings have suggested higher risk of weight gain or becoming overweight or obese among children with higher intake of SSBs [5,6]. However, observational studies are susceptible to unmeasured confounding by socioeconomic position (SEP), unhealthy lifestyle, and health status, making the interpretation uncertain [7]. Meta-analyses of observational studies not corrected for multiple testing or multiple modeling may be biased and unreliable [8]. A few randomized controlled trials (RCT) have been conducted in children [9,10], which suggested reducing SSB intake reduces weight. However, meta-analyses of RCTs have revealed mixed findings. One meta-analysis showed no overall effect of SSB intake on weight in children, possibly due to low compliance in some included trials [6]. This meta-analysis also found weight decrease for reduced sugars intake and weight increase for increased sugars intake in trials of adults with ad libitum diets [6]. However, some others showed small effects of SSB intake on weight or BMI, which may be more marked for increasing consumption of SSB on weight gain and among the overweight [5,11,12]. As a beverage containing naturally occurring sugars, 100% fruit juice was not associated with weight gain in children aged 7 to 18 years in a meta-analysis of observational studies [13], but no RCT to date has tested the effect of 100% fruit juice on adiposity in children.

Given the inconclusive evidence concerning the effect of SSB on adiposity, assessing the association in settings with different confounding patterns can be helpful [14]. Unlike in Western developed settings, obesity is less clearly socially patterned in Hong Kong Chinese children [15,16]. Chinese children also consume fewer SSBs than those in Western developed countries [17]. SSB consumption in Chinese children may have different socioeconomic patterns than in Western settings, where children of lower SEP tend to consume more SSB [18]. Here, we examined the association of SSB frequency at 11 and 13 years with subsequent BMI z-score and overweight (including obesity) up to 18 years and waist circumference (WC), waist-to-hip ratio (WHR) and body fat percentage (BFP) at 16 to 19 years in the non-Western developed setting of Hong Kong using a large, population-representative Hong Kong Chinese birth cohort “Children of 1997”.

## 2. Methods

### 2.1. The “Children of 1997” Birth Cohort

The “Children of 1997” birth cohort is a population-representative Chinese birth cohort (*n* = 8327) that included 88.0% of all births in Hong Kong from April 1, 1997 to May 31, 1997, as described in detail elsewhere [19]. Newborns were recruited at their first postnatal visit to any of the 49 Maternal and Child Health Centers (MCHC) in Hong Kong. Baseline characteristics, including maternal and infant characteristics (birth weight, birth order, maternal age at birth, delivery mode), socioeconomic characteristics (mother’s birthplace, highest parental education), and early life factors (breastfeeding), were assessed using a self-administered questionnaire in Chinese at recruitment. Highest parental occupation, type of housing tenure and household income were also recorded. Passive follow-up via record linkage was instituted in 2005 to obtain all routine annual measurements of height and weight from age 6 years onwards from the Student Health Service (SHS). Height without shoes was measured by stadiometer to the nearest 0.1 cm, and weight without shoes and outer clothing was measured by calibrated digital scales to the nearest 0.1 kg. A postal survey (Survey I) was sent in July 2008, then re-sent a second and third time as necessary to non-respondents over the following 9 months. The survey in Chinese (or English if requested) included questions on food frequency (e.g., fruit, vegetable, meat, SSB), physical activity, general health, main caregiver, and WC (with measuring tape and detailed instruction) of the child. One feature of our setting is that the calculated calorie intakes seemed to be within plausible ranges [20]. A postal survey follow-up (Survey II) was conducted in 2010–2012, which included similar questions on food frequency. The Biobank Clinical Follow-up in 2013–2016 included measurements of height, weight, WC, hip circumference and BFP according to standard protocols. Weight and BFP were measured using bioelectrical impedance analysis (BIA) with a Tanita segmental body composition monitor (Tanita BC-545, Tanita Co., Tokyo, Japan). WC and hip circumference were measured by trained technicians using a standard protocol. Ethical approval for the study, including comprehensive health-related analyses, was obtained from the Institutional Review Board of the University of Hong Kong/Hospital Authority Hong Kong West Cluster (HKU/HA HKW IRB).

### 2.2. Exposure-SSB Consumption

SSB consumption at 11 years was assessed by asking parents in Survey I “On average, how often did your child drink canned pop/soda/soft drinks in the last week?” with responses “never ever drinks” (***n*** = **162**, 4.5%), “not at all in the last week” (***n*** = **1039**, 28.6%), “1–3 times a week” (***n*** = **1841**, 50.7%), “4–6 times a week” (***n*** = **340**, 9.4%), “once a day” (***n*** = **193**, 5.3%), “2–3 times a day” (***n*** = **40**, 1.1%), and “4 or more times a day” (***n*** = **13**, 0.4%). Reponses were combined into the following categories: “less than weekly”, “1–3 times per week”, “4–6 times per week” and “daily” because of the relatively small numbers in some categories. The food frequency questionnaire in Survey I showed some validity regarding the consumption of milk and non-milk dairy products [21]. We also assessed SSB consumption at 13 years by asking children the same question with the same responses in Survey II. We did not assess the consumption of specific types of SSB, therefore the reported intake of SSB included beverages sweetened with sugar and beverages sweetened with non-nutritive sweeteners.

### 2.3. Outcomes–BMI, WC, WHR and BFP

The primary outcomes were age and sex-specific BMI z-scores relative to the 2007 WHO growth reference for 5–19 years [22] at each age from 12 to 18 years. Overweight and obesity were defined using the 2000 International Obesity Task-force (IOTF) cut-offs [23], i.e., overweight is equivalent to an adult BMI of ≥25 to <30 kg/m^2^, and obesity to an adult BMI of ≥30 kg/m^2^. We also calculated BMI z-scores and centiles using the 2012 IOTF reference [24]. Due to the relatively small number regarding obesity (~4%), overweight and obese were combined. The secondary outcomes were WC (cm), WHR, and BFP (%) at 16–19 years (mean age at measurement 17.5 ± 0.5 years).

### 2.4. Statistical Analysis

In the “Children of 1997” birth cohort, we used chi-squared tests and Cohen effect sizes (Cohen’s w effect size for categorical variables, 0.1 for small, 0.3 for medium, and 0.5 for large) [25] to compare participants with and without information on SSB at 11 years. Cohen effect sizes in contrast to chi-squared tests are independent of sample size. We also used one-way analysis of variance (ANOVA) or chi-squared tests to assess the relation of potential confounders with SSBs intake. We used multivariable generalized estimating equations (GEE) with an “exchangeable” (i.e., assuming correlation between measurements were consistent over time) working correlation structure to assess the adjusted associations of SSB consumption at 11 years with BMI z-score and overweight (including obesity) from 12 to 18 years, and we used multivariable linear regression models to assess the associations of SSB consumption at 11 years with WC, WHR and BFP at 16–19 years. Confounders, considered as common causes of SSB consumption and adiposity, were age, sex, maternal age at birth, mother’s birthplace, highest parental education, highest parental occupation, household income per head, main caregiver, general health status, physical activity, and fruit, vegetable and meat consumption and BMI z-score at 11 years. We present four models. Model 1 adjusted for age and sex. Model 2 additionally adjusted for maternal age at birth, mother’s birthplace, highest parental education, highest parental occupation, household income per head and the interaction of mother’s birthplace and highest parental education [15]. Model 3 additionally adjusted for main caregiver, general health status, and physical activity. Model 4 additionally adjusted for fruit, vegetable, and meat consumption and BMI z-score at 11 years.

To handle missing data, we combined multiple imputation (MI) and inverse probability weighting (IPW). We used multiple imputation for missing confounders (among 3300 with exposure and at least one measurement of BMI from 12 to 18 years, maternal age at birth for less than 0.1%, mother’s birthplace for 0.3%, highest parental occupation for 12.8%, household income per head for 11.2%, main caregiver for 0.7%, general health status for 3.2%, physical activity for 11.2%, fruit consumption for 0.3%, vegetable consumption for 0.5%, meat consumption for 0.5%, and BMI z-score at 11 years for 18.4%) based on a flexible additive regression model with predictive mean matching incorporating data on sex, birth order, maternal age at birth, mother’s birthplace, father’s birthplace, highest parental education, highest parental occupation, household income per head, main caregiver, general health status, physical activity, fruit, vegetable, and meat consumption and BMI z-score at 11 years, SSB consumption, and outcomes (BMI z-scores from 12 to 18 years, WC, WHR or BFP). We imputed missing data 20 times using the “Hmisc” R package. We calculated the probability of observations being present using logistic regression models to identify potential predictors of missingness, from which inverse probability weights of response were obtained for each observation. We analyzed the 20 imputed datasets using these weights and summarized the results into single estimated beta-coefficients or odds ratios (OR) with 95% confidence intervals (CI) adjusted for missing data uncertainty [26]. We repeated the analyses by combining MI with inverse probability weighting (IPW) [27]. For comparison, we also conducted a complete case analysis. Given the potential under-reporting of SSB frequency, as a sensitivity analysis we repeated the complete case analyses after randomly reallocating some of those reporting “less than weekly”, “1–3 times per week” and “4–6 times per week” to a higher frequency category to evaluate the effect on the association of SSB consumption with overweight (including obesity). We also assessed the associations of SSB consumption at 13 years with BMI z-score and overweight (including obesity) from 14 to 18 years by using GEE, and with WC, WHR and BFP at 16–19 years by using multivariable linear regression models adjusting for age, sex, maternal age at birth, maternal birthplace, parental highest education level, parental highest occupation, household income per head, and interaction of maternal birthplace with parental highest education level in a complete case analysis.

All statistical analyses were performed using R version 3.6.1 (The R Foundation for Statistical Computing, Vienna, Austria).

## 3. Results

Of the 8327 individuals originally recruited to the “Children of 1997” birth cohort, as of December 2018, 28 had permanently withdrawn. Of the remaining 8299, 7934 were potentially contactable for Survey I in 2008–2009, whilst 75 migrated without trace, 12 deceased, and 278 were untraceable (probably migrated or dead). Of these 7934, 3678 responded to Survey I, of whom 98.6% provided information on SSB consumption at about 11 years (mean age at assessment 11.5 ± 0.3 years) (Figure S1). The proportion of English responses was very low (7/3678 = 0.2%). The 3628 children with information on SSB consumption and 4670 children without were significantly different relating to some characteristics, but the magnitude of the difference was small for sex, maternal age at birth, mother’s place of birth, highest parental education, highest parental occupation, and household income per head (all Cohen effect sizes <0.1) [25] (Table S1). At about 11 years, 33.1% had not consumed SSB during the past week, 50.7% consumed 1–3 times during the past week, 9.4% consumed 4–6 times, and 6.8% consumed daily. The mean of BMI at 11 years was 18.55 ± 3.24 kg/m^2^ and BMI z-score was 0.21 ± 1.27. Table 1 shows that boys, those with younger mothers, mothers born in Hong Kong, or those had ≥1 h/day of physical activity, less frequent fruit and vegetable consumption, and more frequent meat consumption were more likely to consume SSB. SSB consumption was not related to SEP (parental education, parental occupation, and household income).

Table 2 shows that SSB consumption was neither associated with BMI z-score nor overweight (including obesity) from 12 to 18 years in all four models using MI/IPW. Repeating the analysis using a complete case analysis or using BMI z-score or centile based on the 2012 IOTF reference [24] in a complete case analysis produced similar results (Table S2). There were no clear dose-response associations of SSB consumption with WC, WHR or BFP at 16–19 years in all four models using MI/IPW (Table 3) or complete case analysis (Table S3). After considering potential under-reporting of SSB frequency, we found that a positive association of 1–3 times per week of SSB consumption with overweight was possible if 20% or a larger proportion of 1–3 times per week were underreported as less than weekly, or 15% or more of 1–3 times per week, 4–6 times per week and daily were underreported as a lower frequency category (Table S4). We also found no association of SSB consumption frequency at 13 years with measures of adiposity in a complete case analysis (Table 4). Using a composite measure of SSB intake at 11 and 13 years gave a similar interpretation (Table S5).

## 4. Discussion

In this large, population-representative birth cohort of Hong Kong Chinese children, SSB frequency at 11 or 13 years was not associated with subsequent BMI z-score or overweight (including obesity) up to 18 years, nor with WC, WHR or BFP at 16–19 years.

To our knowledge, this is the first study to examine the association of SSB consumption with adiposity using a prospective cohort of Chinese children. Cross-sectional studies in Chinese children [17,28] have shown a positive association of regular SSB consumption with obesity and abdominal obesity in 6–13-year-olds [28] and an association of SSB consumption with abdominal but not general obesity in 6–17-year-olds [17]. Our lack of association of SSB consumption frequency with subsequent BMI z-score or other measures of adiposity differs from an observational study [29] and meta-analyses of observational studies largely from Western settings [5,6,30,31]. A cohort study conducted in US children suggested that BMI z-score was 0.050 higher (95% CI 0.022 to 0.079) for each additional 8 oz/day of SSB consumed at ages 2 to 17 years [29]. The discrepancy between our study and that of Marshall et al. [29] may be because the participants in our study had a much lower SSB intake (6.8% consumed SSB daily at 11 years) than those in that study (median 8 oz/day at 11 years). Notably, Marshall et al. also found a positive association of water/sugar-free beverages with BMI z scores [29], thus confounding may exist. In the US, both SSB intake and adiposity are strongly associated with socio-economic position, which could confound the estimates [15,16]. The social patterning of SSB consumption and of obesity is less marked in our Hong Kong Chinese setting [15], making our observations less open to confounding. Our finding of no association of SSB frequency with subsequent BMI z-score is inconsistent with two RCTs in children, showing that reducing SSB intake reduces weight [9,10]. A possible explanation is that subjects in the two trials had high daily SSB intake at baseline, thus the findings may not apply to individuals with a low SSB intake. Our finding of little association of SSB consumption frequency with adiposity is more consistent with the limited evidence from meta-analyses of RCTs of SSB showing minor effects on obesity [5,6,11,12] which are more pronounced in overweight individuals.

SSB is the largest source of added sugars in US children [32], although the contribution of SSB to added sugars intake has declined and is no longer the largest source in some countries, such as the Germany [33]. SSB may contribute to obesity because sugar in liquid form adds to calories but induces less satiety than in solid form and may weaken any compensatory reduction in subsequent calorie intake and facilitate over-consumption of calories [2]. However, a recent experimental study in mice found no difference in adiposity by sucrose intake but did find differences by fat intake [34], suggesting that fat rather than sucrose drives over-consumption. Observationally, consumption of 100% fruit juice, which may contain as much sucrose as SSB, is unrelated to weight gain in children aged 7–18 years despite slight weight gain in younger children, although this observation has not been confirmed in an RCT [13]. Instead, the observed association of SSB with adiposity in Western populations could be due to modern patterns of sugary/fatty snacking characterized by high SSB consumption, rather to sugar itself [35].

Despite being conducted in a setting where diet has been reported quite accurately in terms of total calories [36], and confounding may be less marked [15], our study has several limitations. First, SSB consumption was obtained by a food frequency questionnaire and may be underreported due to social desirability bias [37]. Non-differential misclassification of SSB frequency usually biases towards the null, which could have obscured a positive association of SSB with overweight if ≥20% of those drinking SSB 1–3 times/week underreported as less than weekly, or ≥15% of those drinking SSB 1–3 times/week, 4–6 times/week and daily were underreported as a lower category. Baseline SSB consumption may subsequently change. However, we found no association of SSB consumption frequency at 13 years with adiposity. Second, we have no information on total energy intake or amount of SSB intake, because we did not collect comprehensive detailed dietary information but only food frequency information. We have no information on the consumption of specific types of SSB, thus the reported SSB may include beverages sweetened with sugar or non-nutritive sweeteners. We assessed only canned SSB, which may have excluded those in bottles or cups and lead to the underreporting of SSB intake. Third, hydration status was not controlled for in the measurement of BFP. However, BFP was measured in the morning in healthy participants after an overnight fast, which likely reduces measurement error due to hydration status, illness, alcohol use or intense exercise. Fourth, only about 40% of the cohort were included, so selection bias is possible. However, those included and excluded were similar and we used IPW to recover the original sample. Fifth, variability in measures of adiposity was limited and few were obese (~4%), potentially reducing power. We only have 80% power to detect an effect size of 0.09 or greater in BMI z-score (i.e., BMI increase of 0.3 kg/m^2^ or greater) for 1–3 times or more per week as compared to less than weekly, thus we cannot exclude a small effect of SSB on adiposity [5,30], or an effect of SSB for overweight and obese children.

SSB consumption is seen as an important target of intervention to combat obesity in Western countries. However, added sugars and SSB intake have declined while obesity has risen steeply over the same time period in Australia, which to some extent challenges the assumption that reduced consumption of SSB can, in itself, help reverse the upward trend of obesity [38]. SSB consumption, as a marker of an unhealthy lifestyle characterized by more energy intake, less exercise, and a poor dietary pattern [39], alone may not dramatically contribute to obesity over time, but rather a constellation of structural, environmental and personal factors that impact long-term weight gain. Still, the overconsumption of SSB remains an important risk factor for the development of obesity in Western children with high SSB intake and strategies targeting SSB as a source of excess energy appear to be prudent [39]. Better understanding of the role of SSB intake in obesity in different populations would ensure policy initiatives are invested in effective targets of intervention.

## 5. Conclusions

In Hong Kong Chinese children with low SSB consumption and less clear socioeconomic patterning of SSB consumption and obesity, SSB consumption frequency was unrelated to adiposity. Although we cannot rule out the possibility of small effects of SSB, especially for overweight and obese children, our study highlights the role of evidence from different social contexts in examining empirically driven hypotheses from long-term economically developed populations.

## Figures and Tables

**Table 1:** Characteristics by sugar-sweetened beverage (SSB) consumption at 11 years for 3628 children in Hong Kong’s “Children of 1997” birth cohort.

Characteristics	Items	*n*	SSB Frequency at 11y (row %)				*p*-Value *
			<weekly	1–3 times/week	4–6 times/week	Daily	
Sex	Girls	1863	37.5	48.2	8.4	5.9	<0.001
	Boys	1765	28.4	53.4	10.4	7.8	
Maternal age at birth	< = 24y	346	27.2	51.7	11.0	10.1	0.04
	25–29y	1129	32.6	51.4	9.6	6.5	
	30–34y	1438	35.2	49.5	9.5	5.8	
	> = 35y	713	32.4	51.9	8.0	7.7	
Mother’s birthplace	Non-HK	1419	38.7	47.1	7.8	6.4	<0.001
	HK	2200	29.5	53.2	10.4	7.0	
Highest parental education	Grade 9 or below	1029	32.4	50.9	9.2	7.5	0.64
	Grade 10–11	1558	32.9	50.3	9.7	7.1	
	Grade 12 or above	1041	34.2	51.2	9.0	5.6	
Highest parental occupation	Professional	842	34.0	51.1	9.6	5.3	0.10
	Managerial	469	34.3	48.4	10.7	6.6	
	Non-manual skilled	918	29.6	54.1	9.4	6.9	
	Manual skilled	518	35.7	46.3	10.8	7.1	
	Semiskilled	318	29.9	52.2	8.8	9.1	
	Unskilled	93	41.9	48.4	6.5	3.2	
Household income ^#^	1st quintile	588	35.5	47.6	8.8	8.0	0.13
	2nd quintile	626	32.9	51.6	9.9	5.6	
	3rd quintile	630	32.7	48.7	11.3	7.3	
	4th quintile	670	29.9	54.9	8.1	7.2	
	5th quintile	699	34.8	50.4	9.7	5.2	
Main care giver	Parents	2985	33.8	49.8	9.3	7.1	0.15
	Grandparents	224	27.7	57.6	10.3	4.5	
	Other	393	31.6	53.2	9.9	5.3	
General health	Good	2927	32.5	51.3	9.1	7.1	0.21
	Average	515	36.9	48.0	10.3	4.9	
	Poor	68	38.2	45.6	8.8	7.4	
Physical activity at 11y	<1 h/d	2282	34.5	49.8	8.9	6.7	0.02
	≥1 h/d	943	29.3	53.7	10.6	6.5	
Fruit consumption at 11y	<weekly	131	35.1	41.2	14.5	9.2	<0.001
	1–3 times	970	27.2	53.2	11.4	8.1	
	4–6 times	667	29.7	54.0	9.6	6.7	
	Daily	1847	37.0	49.2	7.9	5.8	
Vegetable consumption at 11y	<weekly	69	20.3	49.3	14.5	15.9	<0.001
	1–3 times	325	25.8	51.4	12.3	10.5	
	4–6 times	414	28.0	51.7	11.1	9.2	
	Daily	2802	35.0	50.7	8.6	5.7	
Meat consumption at 11y	<weekly	27	51.9	40.7	3.7	3.7	<0.001
	1–3 times	459	40.3	46.4	5.9	7.4	
	4–6 times	662	31.1	57.1	7.6	4.2	
	Daily	2459	32.1	49.9	10.7	7.3	
SSB frequency at 13y	<weekly	902	48.7	44.7	4.3	2.2	<0.001
	1–3 times/week	1279	29.3	56.6	9.5	4.6	
	4–6 times/week	310	18.7	54.2	16.1	11.0	
	Daily	222	16.2	43.7	17.1	23.0	
BMI (kg/m^2^) at 11y	Mean (SD)	2858	18.3 (3.1)	18.7 (3.3)	18.7 (3.4)	18.7 (3.2)	0.04
BMI z-score at 11y	Mean (SD)	2858	0.12 (1.20)	0.25 (1.29)	0.23 (1.34)	0.28 (1.25)	0.04
Weight status at 11y	Normal	2266	35.0	49.9	9.2	5.9	0.06
	Overweight	515	30.1	54.4	9.3	6.2	
	Obese	77	23.4	53.2	16.9	6.5	
WC (cm) at 11y	Mean (SD)	3184	66.4 (8.7)	66.8 (9.1)	67.3 (9.6)	66.9 (9.4)	0.13
BMI (kg/m^2^) at 12y	Mean (SD)	2121	19.0 (3.0)	19.2 (3.4)	19.2 (3.6)	19.2 (3.2)	0.30
BMI z-score at 12y	Mean (SD)	2121	0.13 (1.13)	0.18 (1.27)	0.15 (1.32)	0.24 (1.14)	0.36
BMI (kg/m^2^) at 13y	Mean (SD)	2378	19.6 (3.0)	19.8 (3.5)	19.9 (3.5)	19.9 (3.3)	0.19
BMI z-score at 13y	Mean (SD)	2378	0.07 (1.07)	0.09 (1.22)	0.14 (1.21)	0.15 (1.16)	0.29
BMI (kg/m^2^) at 14y	Mean (SD)	1925	19.9 (2.9)	20.1 (3.4)	20.3 (3.4)	19.9 (2.9)	0.29
BMI z-score at 14y	Mean (SD)	1925	−0.09 (1.02)	−0.07 (1.15)	0.02 (1.21)	−0.07 (1.07)	0.42
BMI (kg/m^2^) at 15y	Mean (SD)	1658	20.4 (3.1)	20.4 (3.4)	20.5 (3.0)	20.3 (3.4)	0.89
BMI z-score at 15y	Mean (SD)	1658	−0.15 (1.04)	−0.17 (1.15)	−0.10 (1.07)	−0.21 (1.14)	0.91
BMI (kg/m^2^) at 16y	Mean (SD)	647	20.3 (2.8)	20.7 (3.4)	20.9 (2.9)	20.5 (3.4)	0.30
BMI z-score at 16y	Mean (SD)	647	−0.29 (1.02)	−0.22 (1.12)	−0.07 (0.94)	−0.27 (1.13)	0.38
BMI (kg/m^2^) at 17y	Mean (SD)	1258	20.6 (3.3)	21.2 (3.8)	21.0 (3.1)	20.6 (3.1)	0.35
BMI z-score at 17y	Mean (SD)	1258	−0.38 (1.08)	−0.23 (1.21)	−0.25 (1.08)	−0.38 (1.09)	0.41
BMI (kg/m^2^) at 18y	Mean (SD)	464	20.8 (3.5)	20.6 (3.4)	20.0 (3.2)	20.5 (2.9)	0.27
BMI z-score at 18y	Mean (SD)	464	−0.45 (1.15)	−0.53 (1.19)	−0.71 (1.15)	−0.53 (1.06)	0.30
WC (cm) at 16–19y	Mean (SD)	2134	71.3 (9.0)	72.4 (9.3)	71.9 (9.1)	71.8 (7.7)	0.23
WHR at 16–19y	Mean (SD)	2128	0.76 (0.06)	0.77 (0.06)	0.77 (0.06)	0.76 (0.05)	0.37
BFP (%) at 16–19y	Mean (SD)	2136	22.3 (8.3)	21.9 (9.2)	21.4 (8.5)	21.4 (9.0)	0.10

BFP, body fat percentage; BMI: body mass index; SD: standard deviation; SSB, sugar-sweetened beverage; WC, waist circumference; WHR, waist-to-hip ratio. ^#^ Mean (SD) for household income per head in quintiles were 1st quintile: HKD 1746 (SD 419), 2nd quintile: HKD 2853 (325), 3rd quintile: HKD 4365 (557), 4th quintile: HKD 6826 (883) and 5th quintile: HKD 14,945 (15,620) (7.8 HKD = 1 USD). * Differences of continuous or categorical variables between SSB frequencies were evaluated by using one-way ANOVA or chi-squared tests, respectively.

**Table 2:** Associations of sugar-sweetened beverage (SSB) consumption at 11 years with body mass index (BMI) z-score and overweight from 12 to 18 years in Hong Kong’s “Children of 1997” birth cohort.

Model	SSB Consumption	*n* ^#^	BMI z-Score			Overweight (Including Obesity) *		
			Beta	SE	95% CI	OR	SE of logOR	95% CI
1	<weekly	1098	Ref			Ref		
	1–3 times/week	1682	0.027	0.043	−0.058, 0.111	1.21	0.10	0.99, 1.46
	4–6 times/week	310	0.041	0.074	−0.105, 0.187	1.16	0.16	0.84, 1.59
	Daily	210	0.042	0.081	−0.118, 0.201	1.05	0.19	0.72, 1.53
2	<weekly	1098	Ref			Ref		
	1–3 times/week	1682	0.027	0.043	−0.058, 0.112	1.21	0.10	0.99, 1.46
	4–6 times/week	310	0.042	0.074	−0.104, 0.188	1.17	0.16	0.85, 1.61
	Daily	210	0.016	0.082	−0.144, 0.176	0.97	0.20	0.66, 1.43
3	<weekly	1098	Ref			Ref		
	1–3 times/week	1682	0.016	0.043	−0.069, 0.101	1.21	0.10	0.99, 1.47
	4–6 times/week	310	0.037	0.074	−0.109, 0.182	1.18	0.17	0.86, 1.64
	Daily	210	0.000	0.081	−0.159, 0.159	0.97	0.20	0.66, 1.43
4	<weekly	1098	Ref			Ref		
	1–3 times/week	1682	0.004	0.021	−0.037, 0.046	1.16	0.14	0.88, 1.52
	4–6 times/week	310	0.037	0.035	−0.031, 0.105	1.05	0.22	0.68, 1.63
	Daily	210	−0.050	0.045	−0.138, 0.037	0.81	0.30	0.45, 1.45

BMI: body mass index; SE: standard error; CI: confidence interval; OR: odds ratio; SSB, sugar-sweetened beverage. All values were derived from GEE models. Model 1 adjusted for age and sex; Model 2 additionally adjusted for maternal age at birth, maternal birthplace, parental highest education level, parental highest occupation, household income per head, and interaction of maternal birthplace with parental highest education level; Model 3 additionally adjusted for main caregiver, general health, and physical activity; Model 4 additionally adjusted for fruit, vegetable, and meat consumption and BMI z-score at 11 years. ^#^ Number of observations with SSB consumption, covariates, and at least one measurement of BMI. * Reference: normal weight.

**Table 3:** Associations of sugar-sweetened beverage (SSB) consumption at 11 years with waist circumference (WC), waist-to-hip ratio (WHR) and body fat percentage (BFP) at 16–19 years in Hong Kong’s “Children of 1997” birth cohort.

Model	SSB Consumption	WC				WHR				BFP			
		*n*	Beta	SE	95% CI	*n*	Beta	SE	95% CI	*n*	Beta	SE	95% CI
1	<weekly	578	Ref			576	Ref			578	Ref		
	1–3 times/week	846	0.85	0.48	−0.09, 1.78	843	0.003	0.003	−0.003, 0.009	845	0.60	0.34	−0.06, 1.26
	4–6 times/week	157	−0.47	0.79	−2.01, 1.07	157	−0.003	0.005	−0.012, 0.007	158	−0.47	0.56	−1.56, 0.62
	Daily	90	0.44	1.01	−1.55, 2.42	90	−0.001	0.006	−0.013, 0.011	91	0.50	0.71	−0.90, 1.90
2	<weekly	578	Ref			576	Ref			578	Ref		
	1–3 times/week	846	**0.97**	**0.48**	**0.03, 1.91**	843	0.003	0.003	−0.002, 0.009	845	0.60	0.34	−0.07, 1.27
	4–6 times/week	157	−0.44	0.79	−1.99, 1.10	157	−0.003	0.005	−0.012, 0.007	158	−0.54	0.56	−1.63, 0.56
	Daily	90	0.37	1.02	−1.63, 2.37	90	−0.002	0.006	−0.014, 0.011	91	0.33	0.72	−1.08, 1.74
3	<weekly	578	Ref			576	Ref			578	Ref		
	1–3 times/week	846	**1.02**	**0.48**	**0.08, 1.96**	843	0.004	0.003	−0.002, 0.010	845	0.63	0.34	−0.03, 1.30
	4–6 times/week	157	−0.39	0.79	−1.93, 1.15	157	−0.002	0.005	−0.012, 0.007	158	−0.50	0.56	−1.59, 0.59
	Daily	90	0.45	1.02	−1.54, 2.44	90	−0.001	0.006	−0.014, 0.011	91	0.39	0.71	−1.01, 1.79
4	<weekly	578	Ref			576	Ref			578	Ref		
	1–3 times/week	846	0.60	0.38	−0.15, 1.35	843	0.002	0.003	−0.004, 0.007	845	0.40	0.26	−0.12, 0.91
	4–6 times/week	157	−0.46	0.62	−1.68, 0.76	157	−0.003	0.005	−0.012, 0.006	158	−0.55	0.41	−1.36, 0.26
	Daily	90	−0.58	0.84	−2.23, 1.08	90	−0.004	0.006	−0.016, 0.008	91	−0.37	0.54	−1.43, 0.69

BFP, body fat percentage; SE: standard error; CI: confidence interval; OR: odds ratio; SSB, sugar-sweetened beverage; WC, waist circumference; WHR, waist-to-hip ratio. All values were derived from multivariable linear regression models. Model 1 adjusted for age and sex; Model 2 additionally adjusted for maternal age at birth, maternal birthplace, parental highest education level, parental highest occupation, household income per head, and interaction of maternal birthplace with parental highest education level; Model 3 additionally adjusted for main caregiver, general health, and physical activity; Model 4 additionally adjusted for fruit, vegetable, and meat consumption and BMI z-score at 11 years.

**Table 4:** Associations of sugar-sweetened beverage (SSB) consumption at 13 years with body mass index (BMI) z-score and overweight from 14–18 years and with waist circumference (WC), waist-to-hip ratio (WHR) and body fat percentage (BFP) at 16–19 years in Hong Kong’s “Children of 1997” birth cohort.

Outcomes	SSB Consumption	*n*	Beta/OR *	SE ^†^	95% CI
BMI z-score	<weekly	924	Ref		
	1–3 times/week	1230	−0.04	0.05	−0.13, 0.06
	4–6 times/week	283	0.04	0.07	−0.11, 0.18
	Daily	180	0.00	0.09	−0.18, 0.18
Overweight	<weekly	924	Ref		
(including obesity) ^#^	1–3 times/week	1230	−0.08	0.12	−0.32, 0.16
	4–6 times/week	283	−0.15	0.20	−0.53, 0.24
	Daily	180	−0.19	0.23	−0.64, 0.27
WC	<weekly	521	Ref		
	1–3 times/week	669	0.11	0.51	−0.89, 1.11
	4–6 times/week	155	0.17	0.80	−1.40, 1.74
	Daily	108	0.89	0.93	−0.93, 2.71
WHR	<weekly	520	Ref		
	1–3 times	667	0.000	0.003	−0.006, 0.006
	4–6 times	153	0.001	0.005	−0.009, 0.010
	Daily	108	0.005	0.006	−0.006, 0.016
BFP	<weekly	521	Ref		
	1–3 times	668	0.20	0.36	−0.50, 0.91
	4–6 times	155	0.41	0.56	−0.68, 1.51
	Daily	108	0.90	0.65	−0.37, 2.17

BFP, body fat percentage; BMI: body mass index; SE: standard error; CI: confidence interval; OR: odds ratio; SSB, sugar-sweetened beverage; WC, waist circumference; WHR, waist-to-hip ratio. Values for BMI z-score and overweight were derived from GEE models, whereas values for WC, WHR and BFP were derived from multivariable linear regression models. All models were adjusted for age, sex, maternal age at birth, maternal birthplace, parental highest education level, parental highest occupation, household income per head, and interaction of maternal birthplace with parental highest education level. ^#^ Reference: normal weight. * Beta for BMI z-score, WC, WHR and BFP and OR for overweight (including obesity). ^†^ Standard error of logOR for overweight (including obesity).

## Supplementary Materials

The following are available online at https://www.mdpi.com/2072-6643/12/4/1015/s1, Figure S1: Participant flow chart in the “Children of 1997” Birth Cohort, Table S1: Characteristics of children with (n = 3628) and without (n = 4670) information on SSB consumption at 11 years in “Children of 1997” birth cohort, Table S2: Associations of SSB consumption at 11 years with BMI z-score, BMI centile and overweight (including obesity) from 12 to 18 years (complete case analysis), Table S3: Associations of SSB consumption at 11 years with WC, WHR and BFP at 16–19 years (complete case analysis), Table S4: Misclassification of SSB consumption at 11 years (complete case analysis), Table S5: Associations of composite SSB consumption at 11 and 13 years with BMI z-score and overweight from 12#x2013;18 years.

## Abbreviations

ANOVA	one-way analysis of variance
BFP	body fat percentage
BMI	body mass index
CI	confidence intervals
GEE	generalized estimating equations
IPW	inverse probability weighting;
IVW	inverse variance weighting
MI	multiple imputation
OR	odds ratios
RCT	randomized controlled trials
SEP	socioeconomic position
SSB	sugar-sweetened beverage
WC	waist circumference
WHO	World Health Organization
WHR	waist-to-hip ratio.
