# Supplementary material for: Association of Sugar-Sweetened Beverage Frequency with Adiposity: Evidence from the “Children of 1997” Birth Cohort
Source: Nutrients. 2020 Apr 7;12(4):1015. doi: 10.3390/nu12041015 (PMC7231010; doi:10.3390/nu12041015)
Supplement: Supplementary file 1 [file nutrients-12-01015-s001.pdf]

Supplementary Material
for
Association of sugar-sweetened beverage frequency with adiposity: evidence from the "Children of 1997" Birth Cohort

Ting Zhang[1], Shiu Lun Au Yeung[1], Man Ki Kwok[1], Lai Ling Hui[1, 2], Gabriel Matthew Leung[1], C. Mary Schooling[1, 3]

1. School of Public Health, Li Ka Shing Faculty of Medicine, The University of Hong Kong, Hong Kong SAR, China
2. Department of Pediatrics, Faculty of Medicine, The Chinese University of Hong Kong, Hong Kong SAR, China
3. CUNY School of Public Health and Health Policy, New York, USA

Corresponding Author
C. Mary Schooling
E-mail: cms1@hku.hk

Supplementary Material

Figure S1. Participant flow chart in the "Children of 1997" Birth Cohort.

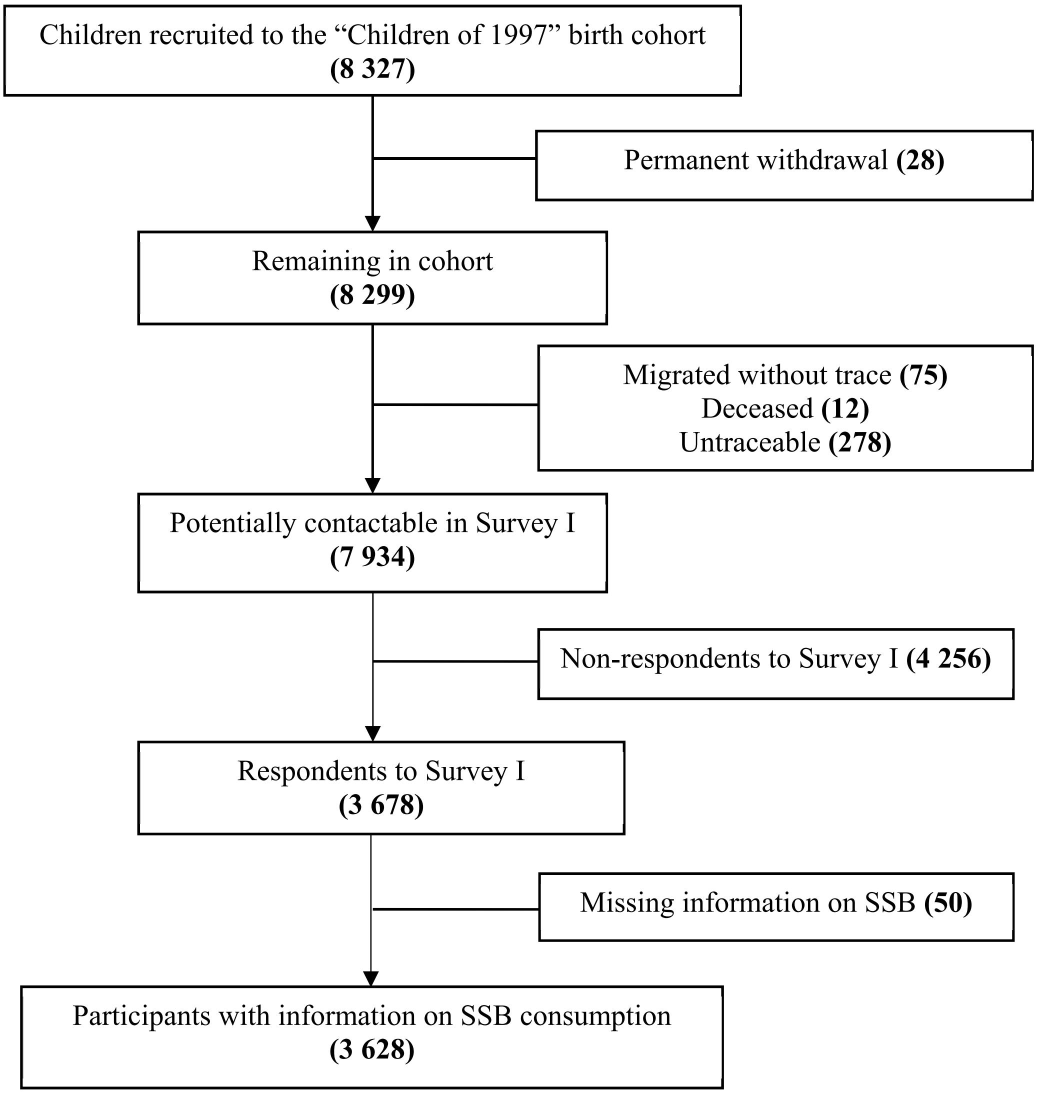

Supplementary Material

Table S1. Characteristics of children with (*n*=3 628) and without (*n*=4 670) information on SSB consumption at 11 years in "Children of 1997" birth cohort.

Characteristics	Items	Children with SSB		Children without SSB		Cohen effect size[1]	P-value[2]
		N	column %	N	column %		
Sex	Girls	1863	51.4	2050	44.2	0.09	<0.001
	Boys	1765	48.6	2584	55.8		
Maternal age at birth	<=24y	346	9.5	662	14.8	0.08	<0.001
	25-29y	1129	31.1	1383	31.0		
	30-34y	1438	39.7	1646	36.9		
	>=35y	713	19.7	767	17.2		
Mother's birthplace	Non-HK	1419	39.2	1633	38.7	0.01	0.66
	HK	2200	60.8	2587	61.3		
Highest parental education	Grade 9 or below	1029	28.4	1438	32.2	0.05	<0.001
	Grade 10-11	1558	42.9	1889	42.3		
	Grade 12 or above	1041	28.7	1142	25.6		
Highest parental occupation	Professional	842	26.7	872	22.3	0.06	<0.001
	Managerial	469	14.9	653	16.7		
	Non-manual skilled	918	29.1	1128	28.9		
	Manual skilled	518	16.4	697	17.9		
	Semiskilled	318	10.1	408	10.5		
	Unskilled	93	2.9	146	3.7		
Household income	1st quintile	588	18.3	863	21.5	0.06	<0.001
	2nd quintile	626	19.5	858	21.4		
	3rd quintile	630	19.6	799	19.9		
	4th quintile	670	20.9	749	18.7		
	5th quintile	699	21.8	741	18.5		
Weight status at 11y	Overweight/obese	592	20.7	801	26.9	0.07	<0.001

1 Cohen effect size has three levels: 0.1 for small, 0.3 for medium, and 0.5 for large. For categorical variables, Cohen's w effect size is calculated as $w = \sqrt(\sum_{i=1}^{N} (P_{0i} - P_{1i})^2 / P_{0i})$ where P_{0i} and P_{1i} are cell probabilities under the null and alternative hypotheses, respectively. Also $w = \sqrt{\chi^2/N}$ where χ^2 is chi-squared statistic and N is the total number of respondents and non-respondents.
2 Chi-squared tests.

Supplementary Material

Table S2. Associations of SSB consumption at 11 years with BMI z-score, BMI centile and overweight (including obesity) from 12 to 18 years (complete case analysis).

Model	SSB consumption	N[1]	BMI z-score (WHO 2007)			BMI z-score (IOTF 2012)			BMI centile (IOTF 2012)			Overweight (including obesity)[2]		
			Beta	SE	95% CI	Beta	SE	95% CI	Beta	SE	95% CI	OR	SE of logOR	95% CI
1	<weekly	1098	Ref			Ref			Ref			Ref		
	1-3 times/week	1682	0.025	0.043	-0.060, 0.109	0.016	0.041	-0.064, 0.095	0.121	1.131	-2.100, 2.340	1.20	0.10	0.99, 1.46
	4-6 times/week	310	0.036	0.074	-0.109, 0.182	0.036	0.070	-0.101, 0.173	0.826	1.908	-2.910, 4.570	1.15	0.16	0.83, 1.58
	Daily	210	0.042	0.082	-0.119, 0.203	0.043	0.078	-0.109, 0.195	0.674	2.212	-3.660, 5.010	1.07	0.19	0.73, 1.56
2	<weekly	867	Ref			Ref			Ref			Ref		
	1-3 times/week	1367	0.016	0.048	-0.078, 0.110	0.004	0.045	-0.085, 0.093	-0.224	1.268	-2.709, 2.261	1.13	0.11	0.91, 1.41
	4-6 times/week	258	0.022	0.084	-0.143, 0.187	0.019	0.079	-0.135, 0.174	0.273	2.140	-3.921, 4.466	1.18	0.18	0.83, 1.68
	Daily	159	0.003	0.092	-0.178, 0.184	-0.005	0.088	-0.177, 0.166	-0.411	2.487	-5.286, 4.464	0.79	0.23	0.50, 1.26
3	<weekly	754	Ref			Ref			Ref			Ref		
	1-3 times/week	1171	0.036	0.051	-0.065, 0.136	0.021	0.048	-0.074, 0.116	0.494	1.351	-2.154, 3.142	1.15	0.12	0.90, 1.46
	4-6 times/week	222	0.026	0.089	-0.148, 0.200	0.018	0.084	-0.146, 0.183	0.238	2.263	-4.197, 4.674	1.16	0.20	0.78, 1.72
	Daily	135	0.056	0.098	-0.136, 0.249	0.041	0.093	-0.141, 0.222	0.542	2.648	-4.647, 5.731	0.92	0.24	0.57, 1.48
4	<weekly	621	Ref			Ref			Ref			Ref		
	1-3 times/week	944	-0.013	0.025	-0.063, 0.037	-0.018	0.024	-0.064, 0.029	-0.587	0.717	-1.990, 0.818	0.87	0.20	0.59, 1.27
	4-6 times/week	172	0.051	0.043	-0.032, 0.135	0.055	0.041	-0.025, 0.135	1.412	1.255	-1.050, 3.870	1.09	0.30	0.61, 1.96
	Daily	97	-0.047	0.055	-0.156, 0.061	-0.058	0.052	-0.161, 0.044	-1.041	1.666	-4.310, 2.220	0.82	0.44	0.35, 1.95

BMI: body mass index; SSB, sugar-sweetened beverage; SE: standard error; CI: confidence interval; OR: odds ratio.
All values were derived from GEE models. Model 1 adjusted for age and sex; Model 2 additionally adjusted for maternal age at birth, maternal birthplace, parental highest education level, parental highest occupation, household income per head, and interaction of maternal birthplace with parental highest education level; Model 3 additionally adjusted for main caregiver, general health, and physical activity; Model 4 additionally adjusted for fruit, vegetable, and meat consumption and BMI z-score at 11 years.
1 Number of observations with SSB consumption, covariates, and at least one measurement of BMI.
2 Reference: normal weight.

Supplementary Material

Table S3. Associations of SSB consumption at 11 years with WC, WHR and BFP at 16-19 years (complete case analysis).

Model	SSB consumption	WC				WHR				BFP			
		N	Beta	SE	95% CI	N	Beta	SE	95% CI	N	Beta	SE	95% CI
1	<weekly	578	Ref			576	Ref			578	Ref		
	1-3 times	846	0.82	0.48	-0.11, 1.75	843	0.003	0.003	-0.003, 0.009	845	0.57	0.34	-0.09, 1.23
	4-6 times	157	-0.41	0.79	-1.96, 1.14	157	-0.003	0.005	-0.012, 0.007	158	-0.44	0.56	-1.53, 0.65
	Daily	90	0.45	1.00	-1.50, 2.40	90	-0.001	0.006	-0.013, 0.011	91	0.50	0.70	-0.87, 1.88
2	<weekly	459	Ref			457	Ref			459	Ref		
	1-3 times	688	1.05	0.54	-0.01, 2.10	685	0.003	0.003	-0.003, 0.009	687	0.78	0.38	0.04, 1.52
	4-6 times	129	-0.18	0.88	-1.91, 1.54	129	0.001	0.005	-0.010, 0.011	130	-0.53	0.62	-1.74, 0.67
	Daily	67	0.35	1.16	-1.93, 2.62	67	-0.003	0.007	-0.017, 0.010	67	0.26	0.82	-1.33, 1.86
3	<weekly	397	Ref			396	Ref			397	Ref		
	1-3 times	581	1.36	0.56	0.25, 2.47	579	0.006	0.004	-0.001, 0.013	580	0.96	0.40	0.18, 1.73
	4-6 times	112	0.37	0.92	-1.43, 2.17	112	0.003	0.006	-0.009, 0.014	113	-0.26	0.64	-1.52, 1.00
	Daily	59	0.77	1.20	-1.58, 3.11	59	0.000	0.007	-0.015, 0.015	59	0.43	0.84	-1.22, 2.08
4	<weekly	321	Ref			320	Ref			321	Ref		
	1-3 times	466	0.49	0.50	-0.48, 1.47	465	0.002	0.004	-0.006, 0.009	465	0.57	0.33	-0.07, 1.21
	4-6 times	88	-0.15	0.81	-1.74, 1.44	88	0.000	0.006	-0.012, 0.012	89	-0.59	0.53	-1.62, 0.45
	Daily	46	-0.27	1.07	-2.36, 1.82	46	0.003	0.008	-0.013, 0.019	46	-0.54	0.70	-1.91, 0.83

BFP, body fat percentage; SE: standard error; CI: confidence interval; OR: odds ratio; SSB, sugar-sweetened beverage; WC, waist circumference; WHR, waist-to-hip ratio. All values were derived from multivariable linear regression models. Model 1 adjusted for age and sex; Model 2 additionally adjusted for maternal age at birth, maternal birthplace, parental highest education level, parental highest occupation, household income per head, and interaction of maternal birthplace with parental highest education level; Model 3 additionally adjusted for main caregiver, general health, and physical activity; Model 4 additionally adjusted for fruit, vegetable, and meat consumption and BMI z-score at 11 years.

Supplementary Material

Table S4. Misclassification of SSB consumption at 11years (complete case analysis).

SSB frequency	N			Overweight (including obesity)[2]		
	Normal weight	Overweight[1]	Total	OR	95% CI	P-value
Observed						
<weekly	607	137	744	Ref		
1-3 times/week	901	260	1161	1.15	0.90, 1.47	0.274
4-6 times/week	176	44	220	1.12	0.75, 1.68	0.578
Daily	103	28	131	0.93	0.58, 1.51	0.779
Non-differential misclassification, 20% of “1-3 times/week” under-reported as “<weekly”						
<weekly	384	72	456	Ref		
1-3 times/week	1124	325	1449	1.36	1.01, 1.83	0.041
4-6 times/week	176	44	220	1.31	0.84, 2.04	0.227
Daily	103	28	131	1.09	0.65, 1.83	0.735
Non-differential misclassification, 15% of “1-3 times/week”, “4-6 times/week” and “Daily” under-reported as “<weekly”, “1-3 times/week” and “4-6 times/week”, respectively						
<weekly	458	93	551	Ref		
1-3 times/week	1015	297	1312	1.33	1.01, 1.75	0.041
4-6 times/week	193	46	239	1.20	0.79, 1.82	0.385
Daily	121	33	154	1.09	0.68, 1.73	0.724
Non-differential misclassification, 30% of “1-3 times/week”, “4-6 times/week” and “Daily” misclassified as “<weekly”, “1-3 times/week” and “4-6 times/week”, respectively						
<weekly	251	32	283	Ref		
1-3 times/week	1205	352	1557	2.38	1.56, 3.65	<0.001
4-6 times/week	181	44	225	1.96	1.15, 3.35	0.013
Daily	150	41	191	2.12	1.23, 3.65	0.007
Non-differential misclassification, 10% (male) and 20% (female) of “1-3 times/week”, “4-6 times/week” and “Daily” under-reported as “<weekly”, “1-3 times/week” and “4-6 times/week”, respectively						
<weekly	446	96	542	Ref		
1-3 times/week	1038	296	1334	1.36	1.04, 1.79	0.027
4-6 times/week	181	42	223	1.16	0.76, 1.78	0.493
Daily	122	35	157	1.22	0.78, 1.93	0.385

OR: odds ratio; CI: confidence interval.
1 Being overweight for at least one age between 12-18 y.
2 Compared with non-overweight, all values were derived from GEE model adjusted for all covariates.

Supplementary Material

Table S5. Associations of composite SSB consumption at 11 and 13 years with BMI z-score and overweight from 12-18 years.

SSB consumption	N[1]	BMI z-score			Overweight (including obesity)[2]		
		Beta	SE	P-value	OR	SE of logOR	P-value
Both <weekly	241	Ref			Ref		
<weekly at 11 but more at 13	237	0.076	0.043	0.080	1.44	0.360	0.313
1-3 times/week at 11 but less at 13	218	-0.016	0.046	0.732	0.82	0.334	0.542
Both 1-3 times/week	395	-0.008	0.040	0.839	0.73	0.319	0.324
1-3 times/week at 11 but more at 13	130	0.028	0.054	0.595	1.43	0.377	0.346
4-6 times/week at 11 but less at 13	85	0.079	0.058	0.169	1.25	0.421	0.600
Both 4-6 times/week	27	0.193	0.092	0.037	1.98	0.700	0.329
4-6 times/week at 11 but more at 13	19	0.012	0.142	0.932	2.65	0.616	0.113
Daily at 11 but less at 13	48	-0.057	0.078	0.463	0.53	0.655	0.338
Both daily	22	0.117	0.095	0.218	0.52	0.785	0.408

OR: odds ratio; SE: standard error.
All values were derived from GEE models adjusted for all covariates.
1 Number of observations with SSB at both timepoints, covariates, and at least one measurement of BMI.
2 Reference: normal weight.
